# The Zebrafish as an Emerging Model to Study DNA Damage in Aging, Cancer and Other Diseases

**DOI:** 10.3389/fcell.2018.00178

**Published:** 2019-01-10

**Authors:** Maria Luisa Cayuela, Kathleen B. M. Claes, Miguel Godinho Ferreira, Catarina Martins Henriques, Fredericus van Eeden, Máté Varga, Jeroen Vierstraete, Maria Caterina Mione

**Affiliations:** ^1^Telomerase, Cancer and Aging Group, Surgery Unit, Instituto Murciano de Investigación Biosanitaria-Arrixaca, Murcia, Spain; ^2^Center for Medical Genetics, Ghent University, Ghent, Belgium; ^3^Institute for Research on Cancer and Aging, Nice, France; ^4^Department of Oncology and Metabolism, Bateson Centre, University of Sheffield, Sheffield, United Kingdom; ^5^Bateson Centre, BMS, University of Sheffield, Sheffield, United Kingdom; ^6^Department of Genetics, Eötvös Loránd University, Budapest, Hungary; ^7^MTA-SE Lendület Nephrogenetic Laboratory, Budapest, Hungary; ^8^CIBIO, University of Trento, Trento, Italy

**Keywords:** zebrafish, DDR, aging, telomeres, cancer, genome maintenance, disease model, p53

## Abstract

Cancer is a disease of the elderly, and old age is its largest risk factor. With age, DNA damage accumulates continuously, increasing the chance of malignant transformation. The zebrafish has emerged as an important vertebrate model to study these processes. Key mechanisms such as DNA damage responses and cellular senescence can be studied in zebrafish throughout its life course. In addition, the zebrafish is becoming an important resource to study telomere biology in aging, regeneration and cancer. Here we review some of the tools and resources that zebrafish researchers have developed and discuss their potential use in the study of DNA damage, cancer and aging related diseases.

## Introduction

Dysfunctional DNA damage repair (DDR) underlies multiple diseases, including age-associated diseases and cancer. The zebrafish, a model organism exploited mostly in developmental studies, is now emerging as a powerful tool to study adult diseases, and scientists are exploring the potentials of this organism for studying how DNA damage dysfunctions impact human health and life span.

Scientists gathering at the 5th European Zebrafish PI meeting held in Trento on 20–23 March 2018 had a dedicated workshop to present their latest research in Aging, DDR and Cancer. This gave them the opportunity to plan the writing of an overview article that summarizes the state of the art in this field. This perspective article conveys our view on the advantages of zebrafish as a model vertebrate organism to study aging, DDR and cancer and sets up the stage for investigating the complex interplay of metabolic, immune and pathophysiological changes associated with these conditions.

In this perspective article, we summarize the recent advances using the zebrafish model for studying diseases with a major DNA damage component. We survey the tools to investigate the impact of DDR dysfunctions on diseases and review the contribution of the zebrafish telomerase mutant to the understanding of aging and its relation with cancer. Moreover, we report how technical advances in zebrafish disease modeling impacted our field, not only by providing more technical resources, but also by furthering our understanding of the mechanisms of gene compensation following mRNA depletion and DNA repair following CRISPR-Cas9 genetic manipulations.

## Perspectives in Studying Aging and Congenital Diseases Linked to Aging and DDR in Zebrafish

Until now, aging research has mainly focused on single diseases. However, this approach does not address the fact that approximately 60% of people over 65 suffer from multiple diseases at the same time, termed multimorbidity (Tinetti et al., 2012; Fabbri et al., 2015). There is, therefore, the urgent need to understand the hallmark mechanisms that go awry with aging and promote and underlie multiple chronic diseases of the elderly.

One important hallmark of aging in humans is telomere dysfunction. Critically short telomeres cause cells to stop dividing, and either die by apoptosis or enter a state of “dormancy” termed senescence. Importantly, senescent cells accumulate aberrantly with aging in multiple organisms, including humans (Dimri et al., 1995), mice (Krishnamurthy et al., 2004), and zebrafish (Carneiro et al., 2016b) and there is strong evidence implicating telomere dysfunction and senescence in age-associated pathologies, such as atherosclerosis (Minamino and Komuro, 2007), arthritis (Price et al., 2002), liver cirrhosis (Wiemann et al., 2002), chronic obstructive pulmonary disease (Amsellem et al., 2011), and cancer (Campisi, 2013).

The zebrafish has recently emerged as a powerful complementary model to investigate the fundamental mechanisms of aging underlying disease. Like humans, and unlike most lab-mice, both naturally aged (Carneiro et al., 2016b) and telomerase mutant zebrafish, accumulate telomere-dependent replicative senescent cells in aged tissues (Anchelin et al., 2013; Henriques et al., 2013; Carneiro et al., 2016b), making the zebrafish telomerase mutant an excellent model of premature aging (see “Telomerase and TR” section below for a detailed discussion of the telomerase mutant).

Zebrafish age-dependent tissue degeneration occurs in a time- and tissue-dependent manner, and, for most tissues, it is anticipated and exacerbated in the absence of telomerase. There are critical tissues that age before others, such as the intestine and muscle, and this is tightly linked to telomere shortening and DNA damage in natural, wild type (WT) aging fish (Carneiro et al., 2016b). Importantly, this is reminiscent of the human scenario, where telomerase loss-of-function mutations, or mutations affecting telomere stability, lead to premature aging syndromes (termed progeria, Hofer et al., 2005; Alter et al., 2012).

In the past decades, the study of human monogenic accelerated aging disorders, such as ataxia-telangiectasia (A-T), Bloom’s syndrome (BS), Cockayne syndrome (CS), Dyskeratosis congenita (DC), Fanconi anemia (FA), Rothmund-Thomson syndrome (RTS), and Werner syndrome (WS), has uncovered some commonalities in their etiology. One recurring theme appears to be impairment of one or more pathways related to DNA repair: RecQ class DNA helicases in BS, RTS and WS (de Renty and Ellis, 2017; Lu et al., 2017; Oshima et al., 2017), the homeostatic protein kinase ATM in A-T (Shiloh and Lederman, 2017) and at least some CS and FA gene products (Brosh et al., 2017; Karikkineth et al., 2017) have all been implicated in the maintenance of genomic stability.

Given the relatively large number of genes involved in monogenic aging disorders it is striking that up to date only a handful of *bona fide* mutations have been characterized in zebrafish: *brca2/fancd1*, *fancl* and *rad51/fancr* (Rodríguez-Marí et al., 2010, 2011; Botthof et al., 2017). Synthetic antisense morpholino (MO) knockdown of *ercc6* (Wei et al., 2015) and *fancd2* (Liu et al., 2003) have been also described, and TALEN and CRISPR mutants for *atm* (Thomas et al., 2014) and *ercc5/xpg* (China Zebrafish Resource Center [CZRC], 2017) have been reported, but not characterized in detail. As for almost all other genes, insertional and ENU mutants already exist at ZIRC and EZRC. Given how straightforward it is to generate novel alleles with CRISPR, it is likely that this apparent dearth of zebrafish aging disorder models will change soon.

A peculiar characteristic of zebrafish as a model for DDR-related conditions is that the impairment of this pathway often results in biased sex ratios. The first documented cases were described in *brca2/fancd1* and *fancl* mutants, both models for FA, where homozygous mutants all developed as males (Rodríguez-Marí et al., 2010, 2011). The sex bias was restored when *tp53* was also blocked, demonstrating that p53-dependent apoptosis has a role in the development of sex bias.

Recently, mutations in 19 genes related to the Fanconi anemia (FA) pathway were created (*fanca, fancb, fancc, fancd1/brca2, fancd2, fance, fancf, fancg, fanci, fancj/brip1, fancl, fancm, fancn/palb2, fanco/rad51c, fancp/slx4, fancq/ercc4, fanct/ube2t, faap100, and faap24*). Knockouts for 12 of these genes showed complete female-to-male sex reversal, and partial sex-reversal was seen in KOs of five more genes. Sex reversal in the case of fancp was Tp53-dependent, just as with the previously reported fancd1, fancl, and fancr mutants. And while mutant males and females were mostly fertile, fancd1, and fancj mutants showed partial and complete sterility, respectively (Ramanagoudr-Bhojappa et al., 2018). These results substantiate the role of the FA-pathway in the PGC-dependent sex determination process of zebrafish and provide further proof for the role of some DDR genes in germ-cell specification.

The germ line has an active role in zebrafish sex-determination (Siegfried and Nüsslein-Volhard, 2008). More precisely, primordial germ cells (PGCs) in the developing juvenile ovary have a key and most likely inductive role in gonad differentiation and their number is a deciding factor in sex-determination. Individuals with lower PGC number become male, whereas high PGC count generally results in females (Tzung et al., 2015). Therefore, the survival and expansion of the PGC population during early phases of development adds another layer of control to the complex polygenic sex determination system, characteristic for zebrafish (Liew et al., 2012).

With this foresight, the sex biased phenotype of *brca2/fancd1* and *fancl* mutants suggests that genes involved in DDR might have an important role for the survival and proliferation of PGCs in the developing gonads. Defects in meiotic recombination do not automatically lead to PGC death, however, for instance *mlh1* mutant females and males are capable of producing eggs and sperm but resulting embryos are aneuploid (Leal et al., 2008). Also in the telomerase mutant, PGCs proliferation is impaired (Anchelin et al., 2013; Henriques et al., 2013) and telomerase has been reported to respond to mild DNA damage with an increased activation (Akiyama et al., 2013).

## Recent Technical Advances in Disease Modeling and Impact on DDR Studies

Previous large-scale mutagenesis approaches, such as the Zebrafish Mutation Project (ZMP) have created mutations in most of the relevant zebrafish genes, and these lines are available from major zebrafish stock centers (^1^Zirc and EZRC). Furthermore, with the adoption of TALEN-based (Sander et al., 2011; Bedell et al., 2012; Reyon et al., 2012) and CRISPR/Cas9-based genome editing technologies (Hruscha et al., 2013; Hwang et al., 2013a,b; Jao et al., 2013; Gagnon et al., 2014; Talbot and Amacher, 2014) for zebrafish, practically any research group can create loss-of-function alleles for the gene(s) of interest.

With regards to DNA repair, it is also noteworthy that over the past couple of years, novel genetic and immunohistochemistry-based tools have been developed that can help to understand the prevalence of different DNA double-strand break (DSB) repair pathways during the repair process, for instance GFP based constructs are available that can report NHEJ, MMEJ, SSA, and HR-based repair (Liu et al., 2012; He et al., 2015).

While during the previous decades of zebrafish research, MOs have been the tool of choice for creating loss-of-function phenotypes (Nasevicius and Ekker, 2000), recently, several studies have highlighted the limitations of this approach (Schulte-Merker and Stainier, 2014; Kok et al., 2015; Stainier et al., 2017). MOs are extremely stable and can be easily delivered into embryos at early stages, where they interfere with translation or splicing. However, the effect of MOs is only transient, therefore they are usually injected in molar excess to have a longer lasting effect. The injection of large amounts of synthetic molecules could be the reason why MOs often elicit strong, specific p53-dependent effects (Robu et al., 2007) and the activation of an innate immune response (Gentsch et al., 2018). Given that DNA damage repair is inseparably linked to p53 activation, it is not surprising that MOs have not been widely adopted in areas of research where DNA damage responses (DDR) play a central role, such as cancer and aging.

*Bona fide* mutants offer a more promising avenue for research modeling diseases related to DDR. One caveat of the genetic engineering approach is that, in zebrafish, mutations caused by genetic engineering manipulations often result in altered mRNA processing (Anderson et al., 2017) or trigger genomic compensation effects through non-sense mediated decay (NMD, Rossi et al., 2015; El-Brolosy and Stainier, 2017) ultimately failing to induce a strong phenotype. New research suggests, however, that these problems can be circumvented with the efficient targeting of the promoter region (El-Brolosy et al., 2018). These data are likely to be particularly useful in the functional analysis of aging-related genes.

## Telomerase: the State of the Art

The telomerase zebrafish mutant has revealed a role for telomeres and telomerase in aging and disease in zebrafish (Carneiro et al., 2016a). Compared to common laboratory mice that possess very long telomeres (40–150 kb), zebrafish telomere length is similar to human telomeres (5–15 kb). Also like humans, telomere length and telomerase expression decrease over time in zebrafish. Telomerase deficient zebrafish (*tert^hu3430/hu3430^ or tert^-/-^*) have shorter telomeres, premature aging phenotype and reduced lifespan. These defects do not occur all at the same time. Strikingly, the majority of tissue dysfunction phenocopies the events occurring during natural zebrafish aging (Anchelin et al., 2013; Henriques et al., 2013; Carneiro et al., 2016b). Over natural aging, the zebrafish intestinal epithelium is one of the first tissues to show gradual DNA damage response activation (53BP1 and γH2AX foci associated with telomeres), increased onset of apoptosis and senescence, and functional defects. Remarkably, telomere shortening in *tert^-/-^* mutants anticipates these alterations in this tissue. However, other proliferative tissues, such as testis or kidney marrow showed altered phenotypes independently of significant telomere shortening. Nevertheless, absence of telomerase has a clear deleterious effect in the adult zebrafish tissues. Thus, in absence of visible telomere shortening in some tissues, lack of telomerase does have a clear impact on their functions, resembling degeneration observed in old age (Carneiro et al., 2016b). This leaves open the hypothesis that, rather than just elongating short telomeres, presence of telomerase may be required for the regenerative capacity of adult organs. In agreement with this idea, telomerase was shown to have a non-catalytic role in hematopoietic cell differentiation (Imamura et al., 2008) and in DDR (Akiyama et al., 2013).

Similarly, also the RNA component (*TERC/TR*) of the telomerase holoenzyme has a non-canonical role in hematopoiesis. DC is a hereditary disease caused by defects in telomere maintenance, and is due to mutations in telomerase components or in telomere-stabilizing components. DC is characterized, in 85% of cases, by cutaneous defects and premature death due to failure in hematopoiesis and immunodeficiency. Mutations in *TERT* (catalytic subunit) and *TERC/TR* (RNA component) are responsible for the dominant autosomic form of DC. Although 90% of patients with DC have problems with the production of three types of blood cells, and all have telomere shortening, the incidence of aplastic anemia (AA), myelodysplastic syndromes (MDS) and cancer is greater in patients with mutations in *TERC/TR*. However, the variability and severity of the symptoms due to the different mutations cannot be accounted for by the sole influence on telomere length (Vulliamy et al., 2011). Indeed, genetic inhibition of *terc* in zebrafish results in neutropenia and monocytopenia (Alcaraz-Pérez et al., 2014), similarly to DC patients. This defect is fully independent of both telomerase activity and telomere length. *Terc/tr* is expressed at very high levels in isolated neutrophils, whereas *tert* is undetectable, suggesting that *terc/tr* has a non-canonical function in these cells. Human *TERC* physically interacts with 2,198 sites throughout the human genome by recognizing the target sequence CCACCACCCC (Chu et al., 2011). These findings suggest that *TERC/TR* has an additional role to that as a telomerase component, and acts as a long non-coding RNA which regulates myelopoietic gene expression, revealing a new target for therapeutic intervention in DC patients.

During aging, short telomeres are deprotected and recognized as DNA damage. As a consequence, both WT and *tert^-/-^* mutants zebrafish accumulate γH2AX foci at telomeres with aging, mainly in gut and muscle (Carneiro et al., 2016b). The formation of γH2AX telomeric foci correlates with telomere shortening, supporting the idea that short telomeres are sensed as DNA damage and activate the DDR. Damaged DNA is recognized by the MRN complex, which recruits the kinases ATM and ATR mediating H2AX phosphorylation. In addition to γH2AX foci, gut and muscles of both aged WT and *tert^-/-^* animals showed a significant reduction of proliferating cell nuclear antigen (PCNA) staining and an increase of senescence markers (e.g., senescence-associated β-galactosidase staining), indicating that telomere shortening leads also to reduction of proliferation and induction of senescence (Anchelin et al., 2013; Henriques et al., 2013; Carneiro et al., 2016b). Decrease of proliferation and accumulation of senescent cells cooperate to the disruption of tissues homeostasis with aging. These effects are mediated by the activation of p53, as the combination of *tert^-/-^* and *tp53^-/-^* mutations rescues the replication rate and partially abolish senescence in the gut (Anchelin et al., 2013; Henriques et al., 2013).

Cells with critically short telomeres rely on either of two mechanisms to avoid cell proliferation. They can either engage a cell death program, through a p53-dependent expression of pro-apoptotic proteins, such as PUMA (Wang et al., 2007), or they can irreversibly arrest the cell cycle, upregulate a second CDKi, *p16Ink4a*, and become senescent (Stein et al., 1999). To date, it remains largely unclear what determines if a cell with short telomeres undergoes senescence or apoptosis. Evidence suggests that most cells are capable of both (Campisi, 2007). Given that both outcomes are initiated by the same type of stimulus and involve the same type of molecular players, this raises the question of how do damaged cells, in an organism, decide whether to continue living in a dysfunctional state or die.

Cancer may result from the occurrence of DNA damage that generates oncogenic events, bypass senescence and apoptosis and activates telomere maintenance mechanisms (TMMs), to allow proliferation and overcome telomere attrition (Reddel, 2014). The study of TMMs in zebrafish cancer models could shed light on the development of this important cancer hallmark. Defining which TMM is adopted by different cancer types, whether overexpression of telomerase (usually linked to mutations or positive regulation at the level of the promoter region) or Alternative Lengthening of Telomeres (ALT), can support diagnosis (mesenchymal and pediatric cancer being more prone to ALT, Apte and Cooper, 2017), prognosis (i.e., Alt+ glioblastomas in adults have a better prognosis, Hakin-Smith et al., 2003) and responses to anti-telomerase therapy (Agrawal et al., 2012). A zebrafish model of brain cancer (Mayrhofer et al., 2017) shows progressive induction of ALT (Idilli et al., unpublished) and recalls the prevalence of this TMM in pediatric brain tumors (Abedalthagafi et al., 2013). One of the hallmarks of ALT is the accumulation of DDR markers at telomeres, which suggests that the repair machinery may have a role in the homologous recombination and chromatid exchange occurring during ALT (Koschmann et al., 2016). Here, the cooperation between the activation of telomere maintenance mechanisms and DNA damage signaling leads to a predominance of surviving and proliferative signals, ensuring the survival and expansion of cancer cells.

## How to Study DDR in Zebrafish?

With the mechanisms and signals of DDR conserved from yeast to mammals, it is not surprising that the reports so far indicate a strict conservation of the molecular players in DDR between zebrafish and humans (see first section). Zebrafish are members of the Teleostei infraclass, and its ancestors underwent an additional round of whole-genome duplication (WGD) called the teleost-specific genome duplication (TSD) (Meyer and Schartl, 1999). Comparison to the human reference genome shows that approximately 71.4% of human genes have at least one obvious zebrafish ortholog and reciprocally, 69% of zebrafish genes have at least one human ortholog (Howe et al., 2013). Interestingly, of the 648 DDR-associated genes, only 70 were found to be duplicated (Supplementary Table S1). Although the zebrafish genome has no *BRCA1* ortholog, it has an ortholog of the *BRCA1-associated BARD1* gene, which encodes an associated and functionally similar protein and a *brca2* gene. Several studies found that zebrafish is a suitable animal model to study DDR.

The DDR response can be studied from within minutes upon DNA damage (recruitment of proteins, foci formation) to hours (residual DNA damage, apoptosis) and days (morphological changes) up to months later (tumor formation). When evaluating the expression of genes involved in the DNA repair system, time kinetics experiments are important – this parameter has to be taken into account and may vary depending on the DNA damage reagent exposed to, or the type of damage to be restored (e.g., Sandrini et al., 2009). We mainly focus here on DNA DSB repair.

Within the first minutes after induction of DNA damage, a wide range of proteins are recruited to the damaged site. This damage can be visualized through immunohistochemistry. Subsequently, after damage recognition, repair proteins are recruited. For example, dividing cells will repair DNA DSB in part through Homologous Recombination (HR), which can be visualized by Rad51 immunostaining. Rad51 foci formation is an important event in HR and defects in genes upstream in this pathway may lead to decrease or even absence of Rad51 foci (Vierstraete et al., 2017).

Unfortunately, there is still a lack of antibodies that can be used reliably in zebrafish, and good DNA repair protein antibodies are rare. Phosphorylated H2AX has been exploited by numerous groups and can be used to visualize and quantify DNA damage (Santoriello et al., 2009; Pereira et al., 2011, 2014; Francia et al., 2012; Drummond and Wingert, 2018), but does not show the repair pathway(s) that are used. There are only sporadic reports on other important DNA repair proteins; Fernández-Díez et al. (2018) published an immunostaining for p53BP1, which promotes non-homologous end-joining-mediated DSB repair while preventing homologous recombination, but signals appeared mainly cytoplasmic, however, Henriques and Ferreira (2012) reported nuclear staining. Here, higher resolution analysis and costaining with Rad51 to confirm the occurrence of HR would be beneficial.

Furthermore pAtm has been detected on tissue sections, but costaining with other DDR proteins has not been shown (Santoriello et al., 2009). Besides immunohistochemical stainings, qPCR, RNAseq and Western blotting provide quantitative information about up- or downregulation of expression and proteins. Although these techniques are (semi-) quantitative, they do not allow visualization of the localization of the damage (e.g., Zheng et al., 2018).

Other assays are “in development,” the van Eeden group fortuitously generated a GFP loss of heterozygosity (LOH) reporter system based on a strong inhibition of Hypoxia Inducible Factor (HIF) by the presence of the Vhl protein (Santhakumar et al., 2012); inactivation of the remaining functional copy of *vhl* in heterozygous (*vhl/+*) cells leads to activation of HIF which in turn activates a sensitive downstream HIF reporter: *phd3:eGFP*. In this system the *vh*l gene is therefore used as a detector of gene damage, where it is expected to be representative of the entire genome, every cell in the embryos that have lost VHL function after a particular treatment will express GFP. This system detects all forms of damage capable of inactivating the gene; chromosome loss, base changes, indels. Thus, it cannot distinguish between different forms of genome stability defects.

Comet assays using larvae have also been reported (Jarvis and Knowles, 2003) as they are popular in toxicology studies. At least one group has used a comet assay to show effects of genetic mutations on levels of DNA damage (Lim et al., 2009). Detailed protocols for several techniques were published by the Amatruda Lab (Verduzco and Amatruda, 2011).

After protein recruitment, repair is performed through either non-homologous end-joining (NHEJ), homologous recombination (HR) and single strand annealing (SSA). Repair through these pathways in zebrafish embryos can be analyzed by utilizing specialized vectors, such as those developed by Liu et al. (2012). Most genetic tools for DSB analysis rely on fluorescence-based systems, where efficient repair following a nuclease-triggered DSB break in the GFP coding sequence leads to functional fluorescent protein. Visualizing efficient repair with such tools is easiest during early stages of development, when essentially all cells can be imaged at once. However, it is important to keep in mind that during these earliest stages of embryogenesis alternative end joining (alt-EJ) is the favored repair mechanism as demonstrated recently by the characterization of *polq* mutants (Thyme and Schier, 2016). Furthermore, although the experiments with these reporter constructs appear technically easy to perform (DNA micro-injection in freshly fertilized eggs) the resulting uneven distribution of the DNA often leads to high variance in the response, and high concentrations of DNA will lead to strong developmental abnormalities.

The lack of good reporter systems for DDR could be alleviated by the use of CRISPR mediated genome modification to tag endogenous proteins involved in DDR, thus circumventing the scarcity of verified antibodies in zebrafish. Unfortunately such precise genome editing has so far been difficult in fish, but novel methods like tethering the repair template to the Cas9 enzyme, using Biotin-Avidin systems (Gu et al., 2018) or Cas9 related enzymes that do not destroy their own binding site (Moreno-Mateos et al., 2017) hold a lot of promise.

Faulty repair of DSBs can lead to the presence of micronuclei in dividing cells, which can be assessed with the micronucleus assay (Pereira et al., 2011; Moreno-Mateos et al., 2017). More micronuclei indicate higher doses of specific DNA damage agents, or higher sensitivity to a specific agent (Oliveira et al., 2009) due to one or more defects in the DDR pathway.

Accumulation of DNA damage might lead to cell death by apoptosis. For this, the TUNEL assay can be applied, on both whole mount zebrafish embryos, as well as sections of embryos or adults. Alternatively, the less sensitive acridine orange assay can be used (Reimers et al., 2006; Usenko et al., 2007).

Defects in the DDR pathway can lead to morphological aberrations in developing embryos. For example, exposure to amifostine will radiosensitize embryos, leading to morphological perturbations during development (Geiger et al., 2006). Interestingly, *tp53* deficiency may revert the effect of irradiation: for example, in irradiated zebrafish embryos *tp53* deficiency does not lead to altered morphological features and actually increases survival, when compared to irradiated wild type controls (Duffy and Wickstrom, 2007).

Accumulation of mutations caused by faulty DDR can lead to genomic instability and ultimately tumorigenesis. For example, *brca2* mutant fish form neoplasia at later stages in life (Rodríguez-Marí et al., 2010). In combination with *p53* deficiency, an accelerated tumorigenesis process can be observed, proving that Brca2 conserved its tumor suppressor role in zebrafish (Shive et al., 2010).

In conclusion, the DDR is a complex process that starts seconds after DNA damage, with effects potentially visible up to months after initial damage. All of the different aspects of DNA damage and (faulty) repair can be observed through a number of different methods in zebrafish. This demonstrates that zebrafish is a powerful system to investigate the DDR response.

## Conclusion and Outlook

Research on DNA damage repair dysfunctions and their impact on aging and cancer is progressing fast and the zebrafish offers an excellent toolbox for these studies, particularly for *in vivo* observation of whole organism effects. Advantages over the mouse system include the similarities in telomere length and maintenance mechanisms (Anchelin et al., 2013; Henriques et al., 2013; Carneiro et al., 2016a), the toolbox for genetic manipulation (reviewed in Mayrhofer and Mione, 2016), the *in vivo* assays, including fluorescent reporters and high throughput chemical screens for drugs affecting DDR.

**FIGURE 1:**
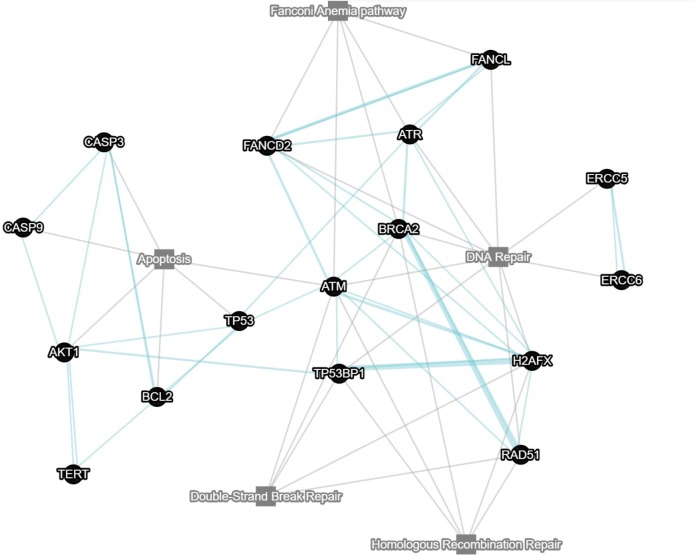
Network displaying key genes involved in apoptosis, DSB repair, Fanconi anemia pathway and homologous recombination. Each node is clickable and linked to a pathway in ZFIN (write to the corresponding author for a hyperlinked version of the figure). Gray lines indicate which pathway a certain gene belongs to. Blue lines indicate pathway associations between genes. Multiple blue lines indicate data from multiple sources. Network was constructed using GeneMANIA.

The number of models (including mutant and reporter lines) is growing (see Figure 1 for a network of DDR-related zebrafish tools, linked to ZFIN pathway database). This is mostly due to the recent addition of somatic transgenics (reviewed in Idilli et al., 2017; Callahan et al., 2018) and somatic knock outs (Ablain et al., 2015; Di Donato et al., 2016). Reporters of DDR and different DNA repair systems are being perfected, while more tools for detection (antibodies, techniques, etc.) are being tested and protocols developed that can benefit our field. Our understanding of the complex relationships between DNA damaged cells and the microenvironment, including the important role of the immune system and damage signals outside of the affected cells is developing and so is our ability to detect and interpret them.

We are confident that zebrafish research will soon be able to provide unique and important contributions to the wider and distinguished community of DDR scholars, with a perspective that embraces molecular events at a tissue level throughout the life-time of a vertebrate.

## Author Contributions

All authors participated in conceptualization and writing of this perspective articles. JV prepared the figure and the table. MM drafted the article and assembled the final version.

## Conflict of Interest Statement

The authors declare that the research was conducted in the absence of any commercial or financial relationships that could be construed as a potential conflict of interest.

## Footnotes

1 Zirc: http://zebrafish.org/fish/lineAll.php, EZRC: https://www.ezrc.kit.edu

## Supplementary Material

TABLE S1 List of human and zebrafish genes involved in DDR, and the corresponding tools (mutant, antibody, etc.) available for zebrafish research. In the second page of the excel file, gene ontology classification of the same gene.
